# Programmed Ribosomal Frameshift Alters Expression of West Nile Virus Genes and Facilitates Virus Replication in Birds and Mosquitoes

**DOI:** 10.1371/journal.ppat.1004447

**Published:** 2014-11-06

**Authors:** Ezequiel Balmori Melian, Sonja Hall-Mendelin, Fangyao Du, Nick Owens, Angela M. Bosco-Lauth, Tomoko Nagasaki, Stephen Rudd, Aaron C. Brault, Richard A. Bowen, Roy A. Hall, Andrew F. van den Hurk, Alexander A. Khromykh

**Affiliations:** 1 Australian Infectious Disease Research Centre, School of Chemistry and Molecular Biosciences, University of Queensland, St. Lucia, Queensland, Australia; 2 Virology, Public and Environmental Health, Forensic and Scientific Services, Department of Health, Queensland Government, Coopers Plains, Queensland, Australia; 3 Division of Vector-Borne Diseases, Centers for Disease Prevention and Control, Fort Collins, Colorado, United States of America; 4 Department of Biomedical Sciences, Colorado State University, Fort Collins, Colorado, United States of America; 5 Queensland Facility for Advanced Bioinformatics (QFAB), University of Queensland, Brisbane, Queensland, Australia; National Institutes of Health,, United States of America

## Abstract

West Nile virus (WNV) is a human pathogen of significant medical importance with close to 40,000 cases of encephalitis and more than 1,600 deaths reported in the US alone since its first emergence in New York in 1999. Previous studies identified a motif in the beginning of non-structural gene NS2A of encephalitic flaviviruses including WNV which induces programmed −1 ribosomal frameshift (PRF) resulting in production of an additional NS protein NS1′. We have previously demonstrated that mutant WNV with abolished PRF was attenuated in mice. Here we have extended our previous observations by showing that PRF does not appear to have a significant role in virus replication, virion formation, and viral spread in several cell lines *in vitro*. However, we have also shown that PRF induces an over production of structural proteins over non-structural proteins in virus-infected cells and that mutation abolishing PRF is present in ∼11% of the wild type virus population. *In vivo* experiments in house sparrows using wild type and PRF mutant of New York 99 strain of WNV viruses showed some attenuation for the PRF mutant virus. Moreover, PRF mutant of Kunjin strain of WNV showed significant decrease compared to wild type virus infection in dissemination of the virus from the midgut through the haemocoel, and ultimately the capacity of infected mosquitoes to transmit virus. Thus our results demonstrate an important role for PRF in regulating expression of viral genes and consequently virus replication in avian and mosquito hosts.

## Author Summary

Programmed ribosomal frameshift (PRF) is a strategy used by some viruses to regulate expression of viral genes and/or generate additional gene products for the benefit of the virus. Encephalitic flaviruses from Japanese encephalitis virus serogroup encode PRF motif in the beginning of nonstructural gene NS2A that results in production of an additional nonstructural protein NS1′ which for West Nile virus (WNV) consists of NS1 protein with 52 amino acid addition at the C terminus. Our previous studies showed that abolishing PFR and NS1′ production attenuated WNV virulence in mice. Here we show by using wild type and PRF-deficient WNV mutant that PRF induces overproduction of structural proteins, which facilitates virus replication in birds and mosquitoes while having no advantage for virus replication in cell lines in vitro. Presence of PRF/NS1′ allowed more efficient virus dissemination in the body of mosquitoes after taking infected blood meal and subsequent accumulation of the virus in saliva to facilitate transmission. Combined with our previous data in mice, the results obtained in this study demonstrate that while having no advantage for WNV replication in vitro, PRF provides advantage for WNV replication in vivo in mammalian, avian and mosquito hosts most likely by overproducing viral structural proteins and generating NS1′.

## Introduction

West Nile virus (WNV) is a flavivirus that circulates in a bird-mosquito enzootic cycle with humans and horses as incidental hosts [1]. It belongs to the Japanese encephalitis subgroup that also includes Japanese encephalitis virus (JEV), St Louis encephalitis virus, and Murray Valley encephalitis virus [1]. The genome of WNV consists of a single-stranded, positive sense mRNA-like RNA molecule of ∼11,000 nucleotides which serves as template for a complementary negative sense RNA. Translation of the positive sense viral RNA produces a single polyprotein that is cleaved during and after translation into 3 structural proteins (C, prM/M, and E) and seven non-structural proteins (NS1, NS2A, NS2B, NS3, NS4A, NS4B, and NS5) [1]. The structural proteins are part of immature and mature virions while the C protein is the sole protein component of the nucleocapsid [1], [2]. Non-structural proteins perform many vital functions of the virus lifecycle including replication (NS1, NS2A) [3]–[7], protein processing (NS3, NS2B) [8]–[10] and virus assembly [11], [12]. Additionally, NS proteins are shown to be involved in modulation of the host cell antiviral responses including inhibition of interferon a/b (IFNα/β) induction (NS2A) [13], IFNα/β/signalling [14]–[18], TLR-3 signal transduction (NS1) [19], and complement activation (NS1) [20].

**Figure 1:**
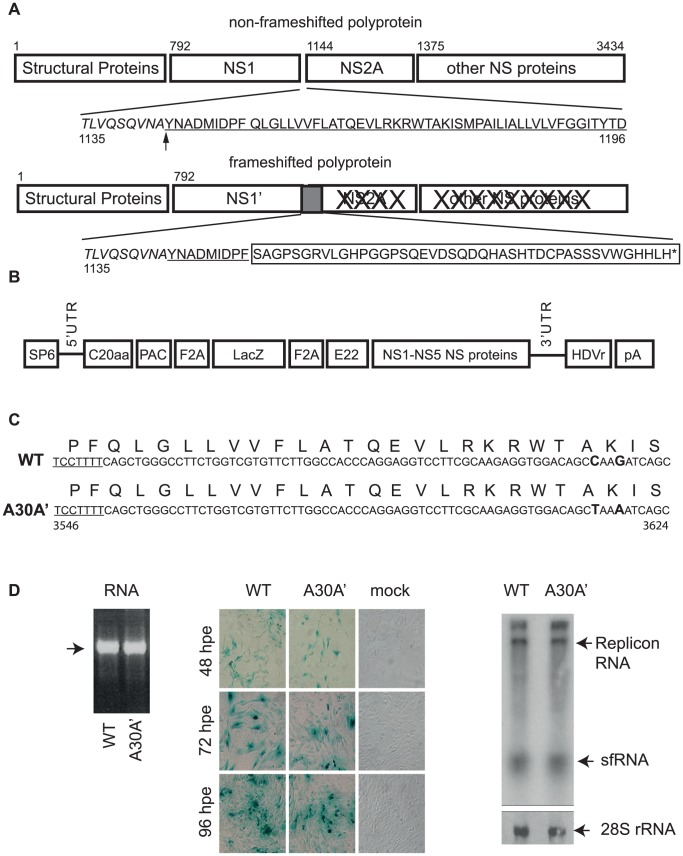
WT and A30A′ replicons show similar rates of replication in BHK cells electroporated with KUNRep-WT or KUNRep-A30A transcribed RNAs. (**A**) Schematic depiction of the translational path of non-PRF and PRF Flavivirus polyprotein. Numbers represent amino acid positions from WNV_KUN_ genome (Accession number AY274504). The underlined sequence represents the start of the NS2A coding region and the box indicates the amino acids encoded by the frameshifted region in NS2A gene. Arrow indicates cleavage between NS1 and NS2A proteins. (**B**) Schematic representation of plasmid DNA encoding WNV_KUN_ replicon KUN-rep. SP6- SP6 RNA polymerase promoter, C20aa – WNV_KUN_ coding sequence encompassing fist 20aa of C gene, PAC- puromycin resistance gene F2A – foot and mouth virus 2A autoprotease, LacZ – β-galactosidase gene, E22 – WNV_KUN_ coding sequence representing last 22 aa of E gene, NS1-NS5 proteins – WNV_KUN_ sequence coding for entire non-structural region, HDVr – hepatitis delta virus ribozyme, pA- polyA signal. (**C**) WNV_KUN_ nucleotide and amino acid sequence in the frameshift region encoded in the WT and A30A′ mutant replicons. The underlined sequence indicates the slippery heptanucleotide frameshift motif and in bold are the two silent nucleotide changes disrupting downstream pseudoknot interactions, introduced in KUNRep-A30A′ replicon. Numbers represent nucleotide positions in WNV_KUN_ genomic RNA. (**D**) *In vitro* transcribed KUNRep-WT and KUNRep-A30A′ RNAs (left panel) was electroporated into BHK cells and expression levels were measured by β-gal staining (48, 72, 96 hpe) (middle panel) and by Northern hybridization (72 hpe) with WNV 3′UTR-specific, ^32^P-labelled DNA probe (right panel).

A feature unique to Flaviviruses in the Japanese encephalitis subgroup is the production of an 11^th^ viral protein; the non-structural protein NS1′. The NS1′ was detected 25 years ago in JEV infected cells [21] but the mechanism of its synthesis was only recently discovered. Firstly, the occurrence of programmed ribosomal frameshift (PRF) in the 5′ terminus of the NS2A gene was established by computational modelling of viral RNA structures by Firth and Atkins [22]. Later, the NS1′ protein synthesis, its amino acid sequence, and RNA sequence requirements for PRF were experimentally demonstrated in mosquito cells, mammalian cells, and cell-free settings [22], [23]. PRF occurred in ∼50% of translational events and led to the production of NS1′ protein containing the entire NS1 sequence, the first 9 aa of NS2A protein, and 43 aa unique to NS1′ (Figure 1A). Translation of NS1′ protein culminated with a stop codon which impeded any further translation in the −1 open reading frame (Figure 1A).

Although the specific function(s) of NS1′ have not been determined several studies had investigated potential roles for NS1′ protein [23]–[27]. We reported that WNV_KUN_ mutants (e.g. A30A′) carrying silent mutations abolishing PRF (and NS1′) without affecting viral accumulation showed attenuated virulence in a mouse model of encephalitis [23]. Interestingly, the JEV strain SA14-14-2 recommended for use as live attenuated vaccine by the World Health Organization contains the single mutation G66A in the NS2A gene which ablated production of NS1′ and reduced neurovirulence and neuroinvasiveness in mice [28]. G66A and G67A mutants in NS2A of another strain of JEV, JaOArS982c, abolishing NS1′ production, have also shown reduced replication in avian DF-1 cells and in embryonated chicken eggs [25]. Interestingly, NS1′ protein produced by the wt JaOArS982 virus was shown to co-localize with NS5 protein [25], indicating a role for NS1′ in RNA replication. Furthermore, Winkelmann et al. demonstrated that mutations eliminating PRF/NS1′ enhanced trans-encapsidation of WNV replicons suggesting a role for NS1′ in virion assembly [26]. Our group previously demonstrated that NS1′ co-localized with NS1 and dsRNA and rescued replication *in trans* of a replication-deficient WNV_KUN_ genome containing large deletion in NS1 gene [27]. More recently studies in other laboratories also showed successful complementation of NS1-deleted WNV genome by the WNV NS1′ protein expressed from an alphavirus replicon vector [29]. These data from different laboratories strongly suggest that NS1′, like NS1, may be one of the components of viral RNA replication complex and therefore plays a role in viral RNA replication.

Despite these insights a clear function for NS1′ remains to be elucidated and some of the results seem to be contradictory. For example, our report that the NS1′-deficient silent mutant A30A′ accumulated to the same levels as WT_KUN_ in C6/36 and mammalian cells seems to indicate that NS1′ is not required for efficient replication and/or virion formation *in vitro*. Additionally, titers of WNV_KUN_ or A30A′ in the serum of infected mice (weanling and adult IRF3^−/−^/7^−/−^) and levels of CPE induced in IFNAR^−/−^ MEFs were similar [23] and unpublished data) suggesting that NS1′ protein may not have an essential function in the viral lifecycle in these models of infection [23], [30].

Moreover, an area of investigation that has been seemingly neglected is the possibility that the frameshift itself has a function in addition to or independent from potential function(s) of the NS1′ protein itself. Since the viral polyprotein is translated as a single molecule (Figure 1A top panel) in the absence of PRF all proteins should be produced in equimolar amounts. It is logical to assume that in the presence of PRF (during WT infection) proteins located upstream of the PRF will be produced in excess to those proteins located downstream of the PRF site (Figure 1A). Since expression of all NS proteins (with the exception of NS1′) is affected by PRF, the viral polyprotein expression event when PRF occurs is essentially separated into structural and non-structural proteins (Figure 1). Conversely, if our hypothesis is correct, in infections with PRF-deficient mutants, such as A30A′ and A30P mutant viruses, all viral proteins would be produced in equimolar amounts and the ratios of structural to non-structural proteins would differ from those observed during WT/PRF-competent infections.

In this context, we decided to perform a wider characterization of the effect of infections with PRF-competent (WT) and PRF-deficient (A30A′ and A30P) viruses. We investigated potential functions of NS1′ protein/PRF such as replication and virion formation during infection of mammalian, avian, and mosquito cell lines. Our studies also included analysis of single nucleotide polymorphisms (SNPs) during infection since SNPs formation may be a manifestation of aberrant replication. We also performed a microarray analysis to determine whether the presence/absence of NS1′/PRF induces significant changes in the transcriptome profile of the infected cells. Importantly, we investigated for the first time the role of NS1′ (PRF) in virus infection, replication, and transmission following infection of *Culex* mosquitoes, the primary vectors of WNV [31] and virus replication following infection of house sparrows, an experimental model of WNV infection in avian hosts [32], [33].

## Results

### Deficiency in PRF/NS1′ does not affect WNV_KUN_ replicon RNA replication

Previously we did not detect significant differences in accumulation of WT and A30A′ WNV_KUN_ viruses in the supernatant of infected Vero 76 or C6/36 mosquito cells after performing multiple cycle growth curves (MSGC) [23]. However, our recent findings demonstrated that NS1′ and NS1 co-localise within infected cells and more importantly that NS1′ protein is able to complement replication of replication-deficient NS1-deletion mutants in trans [27]. These findings suggest a potential role in replication for the NS1′ protein and therefore we decided to employ replicons which allow focusing on RNA replication and exclude processes of virion formation and/or secretion.

To this end we used 2 WNV_KUN_ replicons, SP6KUNrep3βgal (PRF-competent, wt) and SP6KUNrep3βgal/NS2A-A30A′(PRF-deficient). The latter was constructed by replacing the *Bsr*GI 7905–9192 fragment of SP6KUNrep3βgal with the *Bsr*GI 6327–7614 fragment from FLSDX/NS2A-A30A′. This fragment contains two silent nucleotide mutations in the NS2A gene (genome positions 3615 and 3618 C to T and G to A, respectively) resulting in retention of Alanine at position 30 and Lysine at position 31 of the NS2A protein). These mutations disrupt formation of pseudoknot structures required for PRF but do not change the amino acid sequence of the NS2A protein (Figure 1 B and C) [23]. These WNV_KUN_ replicons were named Rep-WT and Rep-A30A′, respectively.

BHK cells were electroporated with similar amounts of *in vitro* transcribed RNAs (Figure 1D left panel) from Rep-WT or Rep-A30A′ replicons and X-gal staining to detect β-gal expressing cells (as means of detecting replicon RNA replication and accumulation) was performed at 48, 72, and 96 hours post-electroporation. X-gal staining of electroporated cells showed similar numbers of β-gal expressing cells at all time-points tested for both replicons (Figure 1D central panel) indicating similar levels of replication for Rep-WT and Rep-A30A′ constructs. These results were confirmed by Northern blot of total RNAs from electroporated cells using a radiolabelled 3′-UTR probe (Figure 1D right panel). Overall, these findings indicate that absence/presence of NS1′/PRF does not have a significant effect on the replication and accumulation of replicon RNAs confirming previous results with full length infectious clones [23].

### Deficiency in PRF/NS1′ does not affect WNV_KUN_ replication and/or spread in different cell lines

A recent publication by Winkelmann et al. suggested a role for NS1 and NS1′ in virion assembly [26]. Using serially passaged WNV/Dengue or WNV/JEV chimeric replicons the authors concluded that a mutation in the frameshift motif in NS2A was selected to eliminate PRF; genomes that were deficient in PRF and failed to make NS1′ (similar to A30A′ and A30P mutants in this work) were more efficiently packaged than WT genomes that were competent in PRF and producing NS1′ [26]. Considering that we did not observe differences in viral accumulation in the supernatant of infected cells or β-gal activity in cells transfected with PRF-deficient replicons, we decided to investigate whether the viral spread was affected by PRF-eliminating mutations by using IFA of cells infected at low MOI.

**Figure 2:**
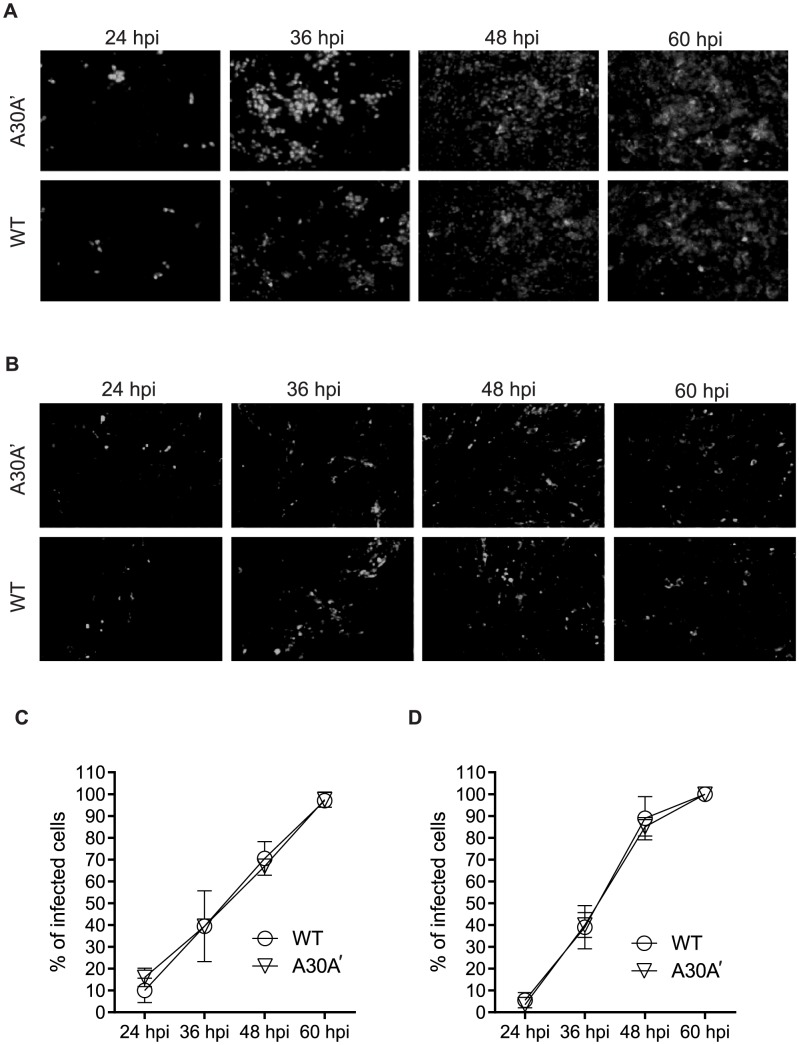
WNV_KUN_ WT and WNV_KUN_ A30A′ viruses spread to neighbouring cells at similar rates. Vero (**A,C**) and BHK cells (**B,D**) were infected at MOI 0.01 with WT or A30A′ viruses. The kinetics of infection was followed by IFA with anti-E monoclonal antibodies [56] at 24, 36, 48, and 60 hpi. The percentage of infected cells was determined automatically by Incell-1000 Workstation software using the equation [(number of cells @480 nm)/(number of cells @ 360 nm)]_KUNV_×100−[(number of cells @480 nm)/(number of cells @ 360 nm)]_mock_×100. Shown are results from two independent experiments.

Vero 76 and BHK cells were infected at MOI of 0.01 with WT or A30A′ WNV_KUN_ viruses and progress of infection was followed by IFA with E-protein specific antibodies. We used a low MOI to ensure a small number of infected cells at early time points and measured the spread of the virus over time. At 24 hpi only a few cells were infected with either virus but by 36–48 hpi most cells were infected (Figure 2 A and B). Measurement of the percentage of cells positive for E-protein at different time points indicated that both viruses spread at a similar rate in Vero 76 and BHK cells (Figure 2 C, D). Additionally, we did not observed any variation in the fluorescent signal intensity (Fig. 2A and B) indicating that both viruses accumulated to similar levels within the infected cells. These findings indicate that a role for PRF in virion formation, or secretion, or spread during viral infections seems unlikely.

We also analysed replication and cytopathicity (CPE) of WT and A30A′ WNV_KUN_ viruses in another mammalian cell line LLC-MK2, derived from African green monkey kidney, as well in immortalized chicken embryo fibroblasts DF-1 to determine whether PRF/NS1′ have any effect on virus replication these experimental systems. In addition to A30A′ mutant we also included A30P virus because this mutation also eliminates PRF and production of NS1′; in addition it also changes amino acid 30 in NS2A protein from Ala to Pro. This mutant has been extensively characterized by us previously and has been shown to induce reduced CPE in various mammalian cells including mouse embryonic fibroblasts and reduced virulence in mice [13], [30], [34].

**Figure 3:**
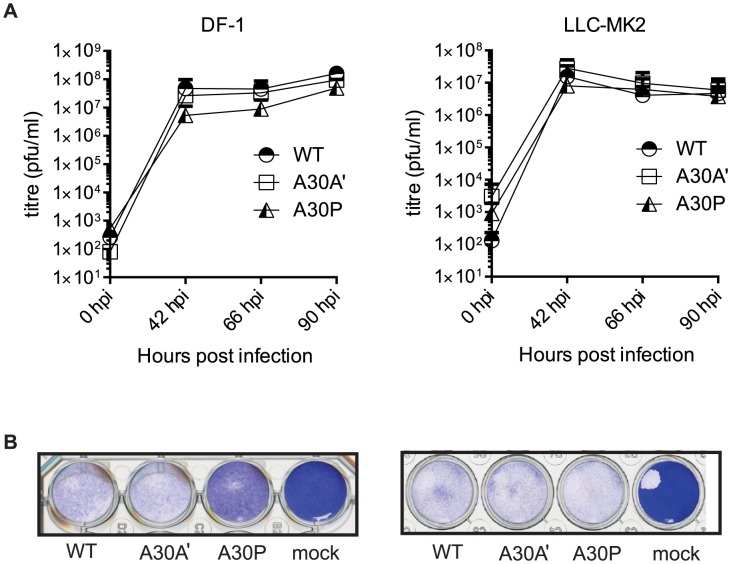
PRF does not impact viral accumulation in avian and mammalian cell lines. DF-1 and LLC-MK2 cell lines were infected with WNV_KUN_ WT, WNV_KUN_ A30A′, and WNV_KUN_ A30P viruses at MOI of 1 and (**A**) Viral titres measured by plaque assay at 0, 42, 66, and 90 hpi. Error bars represent standard deviation. (**B**) Cytopathic effect illustrated by crystal violet staining at 5 dpi of DF-1 and LLC-MK2 cells infected with WT, A30A′, or A30P viruses at MOI of 1. Shown is a representative staining of two independent experiments.

A30P WNV_KUN_ virus showed delayed replication in DF-1 cells compared to WT and A30A′ WNV_KUN_ viruses (Fig. 3A) and significantly reduced CPE even at lower viral loads (MOI = 1 in Figure 3B left pane; MOI = 0.1 data not shown) indicating that the attenuated phenotype of the A30P virus is a characteristic feature of this mutation in the NS2A gene. WT and A30A′ infections however, were indistinguishable and both viruses caused significant and similar severity of CPE upon infection of DF-1 cells (Figure 3A–B). This is in contrast to significant differences between wt and PRF-deficient virus replication in DF-1 cells reported for JEV [25]. It is not unreasonable to assume that PRF deficiency may affect replication of JEV and WNV differently.

In contrast to DF-1 cells, A30P WNV_KUN_ virus showed significant CPE in LLC-MK2 cells and was indistinguishable from WT and A30A′ WNV_KUN_ viruses in growth kinetics and CPE (Fig. 3A–B right panels). Again, WT and A30A′ viruses were indistinguishable in growth properties and in CPE also in LLC-MK2 cells demonstrating no apparent role for PRF in virus replication and virus-induced CPE *in vitro*.

### Global gene expression in MEFs infected with WT, A30A, and A30P WNV_KUN_ viruses

Next we decided to investigate global expression pattern of primary mouse embryonic fibroblasts (MEFs) infected with WT and PRF-deficient WNV_KUN_ mutants to detect potential variations in the host gene expression that may be related to PRF or mutated NS2A protein. We selected WT and IRF3^−/−^/7^−/−^ knockout MEFs because of our previous studies indicating that WNV_KUN_ mutants A30A′ and A30P were attenuated in WT weanling mice while WNV_KUN_ mutant A30P was also attenuated in IRF3^−/−^/7^−/−^ adult animals. This raised the possibility that interferon-independent pathways may be involved in this attenuation, at least for A30P virus [13], [23], [30], [34].

**Figure 4:**
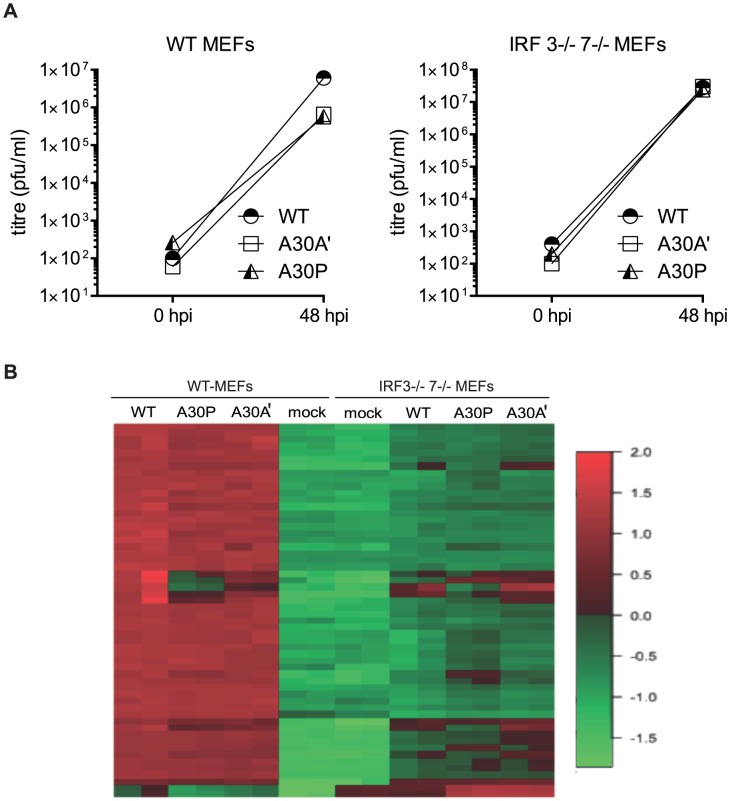
Infection of WT and IRF3^−/−^7^−/−^ MEFs with PRF-deficient WNV_KUN_ viruses induces differential expression of several genes. Low passage primary WT and IRF3^−/−^7^−/−^ MEFs were infected at MOI of 1 with WNV_KUN_ WT, WNV_KUN_ A30A′, or WNV_KUN_ A30P viruses and 48 hpi total RNA was extracted and used for analysis of global gene expression by microarrays. (**A**) Virus titres in the infected cell culture supernatants were assayed by plaque assay at 0 and 48 hpi. (**B**) Heat map showing top differentially expressed genes ranked by log fold change between different experimental conditions.

After infection of WT and IRF3^−/−^/7^−/−^ MEFs with WT, A30A′, or A30P WNV_KUN_ viruses at MOI 1 total RNA was extracted 48 hpi and global gene expression was measured by microarrays. The titers of viruses accumulated in the supernatant of infected cells at this time point indicated that A30P and A30A′ viruses reached lower titers than WT virus during infection of WT MEFs but titers of all viruses in IRF3^−/−^/7^−/−^ MEFs were similar (Figure 4A). As expected the expression of several hundred of genes was affected by viral infection of WT MEFs with 368 genes showing upregulation and 169 genes showing down regulation when compared with non-infected cells (Figure 4B and Table 1). Similarly, after infection of IRF3^−/−^/7^−/−^ MEFs with WT virus, 1639 and 1665 transcripts were upregulated and down regulated, respectively (Table 1). These results indicate that WNV_KUN_ infection induces profound changes in the transcriptome of murine cells and these changes are reflected across numerous pathways.

**Table 1:** Summary of microarray analysis of global gene expression in MEFs infected with WT and mutant WNV_KUN_ viruses.

Cell Type	Comparison	Upregulated	Downregulated
WT MEF	WT vs Mock	368	169
WT MEF	A30A′ vs Mock	318	114
WT MEF	A30P vs Mock	304	81
WT MEF	WT vs A30A′	15	5
WT MEF	A30A′ vs A30P	0	0
WT MEF	WT vs A30P	8	0
IRF3^−/−^/7^−/−^ dko	WT vs Mock	1639	1665
IRF3^−/−^/7^−/−^ dko	A30A′ vs Mock	1470	1528
IRF3^−/−^/7^−/−^ dko	A30P vs Mock	488	366
IRF3^−/−^/7^−/−^ dko	WT vs A30A′	0	11
IRF3^−/−^/7^−/−^ dko	A30A′ vs A30P	305	385
IRF3^−/−^/7^−/−^ dko	WT vs A30P	439	678

Infection with WT or A30A′ WNV_KUN_ viruses in WT or IRF3^−/−^/7^−/−^ MEFs produced similar transcriptional profiles and showed that only a small number of genes were differentially affected between these two infections (Tables 1, 2 and 3). For example, in comparison of WT vs A30A′ infection, 15 transcripts were upregulated and 5 transcripts downregulated in infected WT MEFs (Table 1) and the differences detected were relatively small (1.4–2 fold, Table 2) despite ∼10-fold difference in the viral titres (Fig. 3A). Infection of IRF3^−/−^/7^−/−^ MEFs also resulted in little difference in transcript regulation between WT and A30A′ viruses (Table 1) and again the differences although significant, were small (Table 3). The data indicate that frameshift per se doesn't appear to have a significant effect on host response to WNV_KUN_ infection in MEFs.

**Table 2:** Selected genes differentially up-regulated in WT MEFs infected with WT and A30A′ mutant WNV_KUN_ viruses.

WT vs A30A′		
ID	logFC	Fold
Mmp10	0.94	1.92
Serpinb2	0.74	1.67
Mmp13	0.69	1.61
Gas7	0.68	1.61
Tlr2	0.65	1.57
Cd40	0.63	1.55
1200002N14Rik	0.62	1.53
Atm	0.58	1.49
Clec4e	0.58	1.49
Oasl1	0.57	1.49
Serpina3f	0.57	1.48
Cxcl10	0.56	1.48
Ccrl2	0.53	1.44

**Table 3:** Selected genes differentially up-regulated in IRF3^−/−^/7^−/−^ MEFs infected with WT and mutant WNV_KUN_ viruses.

WT vs A30A′			WT vs A30P			A30A′ vs A30P		
ID	logFC	Fold	ID	logFC	Fold	ID	logFC	Fold
Dus4I	0.98	1.97	Egr4	3.61	12.22	Egr4	3.53	11.58
Hspb1	0.76	1.70	Traf1	3.38	10.40	Traf1	3.27	9.66
Creld2	0.75	1.68	Hspb1	3.13	8.78	Csf2	2.92	7.58
LOC100047583	−0.75	0.59	Rgs16	2.97	7.84	Rgs16	2.83	7.10
Cxcl1	−0.75	0.59	Soat2	2.90	7.49	Soat2	2.71	6.52
Usp18	−0.76	0.59	Csf2	2.72	6.59	Axud1	2.61	6.12
D14Ertd668e	−0.77	0.59	Ngfb	2.63	6.18	Ngfb	2.49	5.63
6330406l15Rik	−0.78	0.58	Lif	2.52	5.75	Hspb1	2.37	5.17
Oasl2	−0.78	0.58	Axud1	2.52	5.72	Lif	2.36	5.14
Itm2a	−0.79	0.58	Ccdc68	2.51	5.71	Ccdc68	2.34	5.05
Nid1	−0.79	0.58	Zswim4	2.39	5.25	Serpinb2	2.33	5.04
Sparc	−0.81	0.57	Serpinb2	2.27	4.82	Arc	2.17	4.51

In contrast, differentially expressed genes were abundant when comparisons included transcriptional changes induced by A30P WNV_KUN_ virus infections but only on the background of IRF3^−/−^/7^−/−^ MEFs (Table 3). The A30P mutation played a significant role in the differential regulation of hundreds of genes compared to the WT or A30A′ virus infections in IRF3^−/−^/7^−/−^ MEFs (Tables 1 and 3). Surprisingly, little or no difference in the host gene transcription levels was detected between WT and A30P infections or between A30A′ and A30P infections in WT MEFs (Table 1). All 3 viruses, however, did induce upregulation and downregulation of a large number of host transcripts in both cell types when compared to Mock cells (Table 1) suggesting that MEFs mount significant antiviral response upon infection with these viruses.

### Single nucleotide polymorphisms in WT and A30A′ WNV_KUN_ genomes

To investigate whether the presence or lack of PRF may affect selection of predominant virus population during infection, we analysed WT and A30A′ viral genome pools for the presence of single nucleotide variants by sequencing using the Ion torrent sequencing platform (see Material and Methods). After quality control of the raw sequence data using fastq, trimmomatic and QFAB proprietary methods, high quality reads were mapped to the reference genomes of WT and A30A′ WNV_KUN_ viruses. Table 4 shows a list of variants detected within the WT (15/52) and A30A′ (15/51) sequence collections and represent rare variants (1% or more) where reads were observed on both sequence strands and were observed in a least 25 independent sequence reads.

**Table 4:** Ion Torrent sequencing analysis of variants in WT and A30A′ mutant WNV_KUN_ virus populations.

Reference	Position in Genome	Reference	Depth	Variant	{Fwd,Rev}	Proportion
WT	218	g	3884	g→A	<4,53>	0.015
WT	253	t	2341	t→G	<37,7>	0.019
WT	835	a	2662	a→C	<2,49>	0.019
WT	947	t	3689	t→G	<2,48>	0.014
WT	949	g	4185	g→T	<2,60>	0.015
WT	950	t	4206	t→A	<1,58>	0.014
WT	951	a	4198	a→T	<0,58>	0.014
WT	1201	g	2411	g→A	<27,1>	0.012
WT	1203	a	2812	a→G	<29,1>	0.011
WT	1261	a	2790	a→G	<0,28>	0.010
WT	1475	c	3408	c→A	<97,3>	0.029
WT	1479	c	3468	c→G	<49,4>	0.015
WT	1480	g	4790	g→C	<46,4>	0.010
WT	1784	a	2670	a→G	<61,1>	0.023
WT	2360	t	3939	t→C	<29,27>	0.014
A30A′	218	g	4499	g→A	<12,76>	0.020
A30A′	253	t	3151	t→G	<59,6>	0.021
A30A′	826	t	3780	t→C	<0,38>	0.010
A30A′	830	t	3774	t→G	<0,38>	0.010
A30A′	831	a	3171	a→T	<0,39>	0.012
A30A′	835	a	3007	a→C	<1,36>	0.012
A30A′	1201	g	2552	g→A	<42,3>	0.018
A30A′	1203	a	2896	a→G	<43,3>	0.016
A30A′	1475	c	4162	c→A	<128,2>	0.031
A30A′	1479	c	4145	c→G	<89,5>	0.023
A30A′	1480	g	5840	g→C	<87,4>	0.016
A30A′	1481	g	5829	c→G	<87,1>	0.015
A30A′	1483	g	5624	g→C	<77,2	0.014
A30A′	1784	a	2875	a→G	<55,2>	0.020
A30A′	2820	c	2241	c→T	<3,50>	0.024

We did not find significant differences between WT and A30A′ WNV_KUN_ genomes related to the presence or abundance of variants (Table 4) with the exception of the two nucleotide mutations introduced in the A30A′ genome by mutagenesis which obliterate PRF formation [23] (positions 3615 and 3618) (Table 5). However, we did find that ∼11% of the WT reads across the pseudoknot region contained a variant at the critical 3615 position, similar to A30A′ mutant (underlined in Table 5), suggesting that WT genome pool does contain mutated genomes unable to elicit PRF events.

**Table 5:** Variations at nucleotide positions 3615 and 3618 in WT and A30A′ WNV_KUN_ virus populations.

Sample	Reference	Position	Reference	Depth	Variant	{Fwd,Rev}	Proportion
WT Chip	A30A′	3615	T	3742	T→C	≤2337,991≥	0.889
WT Chip	A30A′	3618	A	3813	A→G	<2350,1431>	0.992
A30A′ Chip	WT	3615	C	3742	C→T	<2954,2387>	0.999
A30A′ Chip	WT	3618	G	3813	G→A	<2942,2416>	0.991

### PRF alters ratio of structural to non-structural proteins

Studies on PRF in the NS2A gene of flaviviruses have so far focused on the presence/absence of NS1′ and its possible functions on replication, virion formation/secretion, and neuroinvasiveness [23]–[26], [28], [35]. An unexplored consequence of PRF in the NS2A gene is its potential impact on altering the expression of viral proteins. Because Flavivirus proteins are produced from a large single polyprotein, all viral proteins in the absence of PRF should be produced in equimolar amounts. We assumed that occurrence of PRF at the start of the NS2A gene should change this 1∶1 ratio and should produce a surplus of structural proteins. That is, proteins located in the viral genome before the PRF site will be produced from each translatable molecule while proteins located after the PRF will be only produced in molecules not undergoing PRF (Figure 1A). In fact, PRF affects also NS1 protein synthesis (located upstream of PRF) since molecules producing NS1′ do not produce NS1 due to the deficiency in NS1/NS2A cleavage [27]. As a consequence, the occurrence of PRF should affect translation of all non-structural proteins and cause a de-facto change in the ratio of structural to non-structural proteins during translation of the Flaviviral genome (Figure 1A).

To investigate the hypothesis that PRF regulates the ratio of structural to non-structural proteins, a replicon system where the structural genes were replaced by β galactosidase (β-gal) gene was employed. Replacing secretable structural proteins with cytoplasmic β-gal eliminates likely underestimation of structural protein levels when calculating the protein expression ratio due to the secretable nature of structural proteins. Semi-quantitative Western blot was performed to measure the accumulation of β-gal protein and NS proteins (NS3 and NS5) in cells electroporated with WNV_KUN_ Rep-WT and Rep-A30A′ RNAs. If the working hypothesis is correct, the accumulation of NS3 or NS5 proteins relative to β-gal accumulation should be increased in Rep-A30A′ transfected cells, leading to decreased β-gal/NS3 and β-gal/NS5 ratios relative to Rep-WT transfected cells.

**Figure 5:**
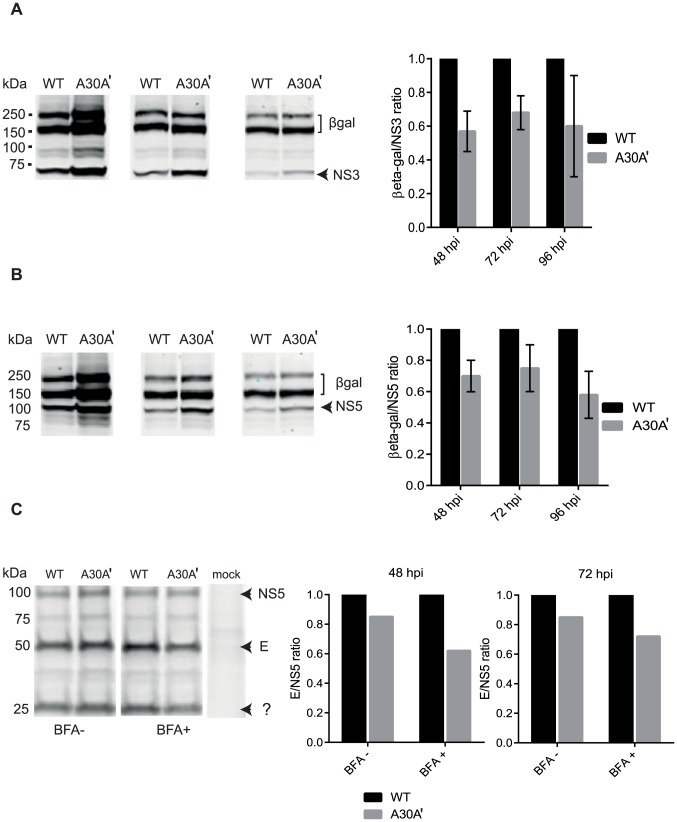
PRF disrupts equimolarity of viral protein synthesis in replicon-transfected and virus-infected cells. (**A**) KUNRep-WT and KUNRep-A30A′ replicons were electroporated into BHK cells and 48, 72, 96 hpe lysates were harvested and total proteins were separated by SDS-PAGE and detected by Western blot with anti-β-gal and anti-NS3 or (**B**) anti-β-gal and anti-NS5 antibodies. Ratios of β-gal/NS proteins were calculated and plotted as a function of KUNRep-WT ratios. (**C**) BHK cells were infected with WNV_KUN_ WT or WNV_KUN_ A30A′ virus and at 48 and 72 hpi proteins were pulse-labelled with ^35^S methionine/cysteine with and without brefedrin A (BFA) treatment, harvested with RIPA buffer and immuno-precipitated with anti-E and anti-NS5 antibodies. Precipitated proteins were separated by SDS-PAGE and visualised by exposure to X-ray film. Ratios of E/NS5 were calculated as above and represent 2 independent experiments. Error bars represent standard error of the mean of 2 independent experiments.

Electroporated cells were lysed with RIPA buffer at 48, 72 and 96 hours post electroporation. Total protein lysates were separated by SDS-PAGE and Western blot analysis was performed with anti-β-gal, anti-NS3, and anti-NS5 antibodies (Figure 5 A, B). The integrated intensities of each targeted protein was determined and ratios of β-gal/NS proteins were calculated for Rep-A30A′ and Rep-WT lysates. For the clarity of presentation, ratio of β-gal/NS proteins in Rep-WT were set to 1.0 so that the β-gal/NS proteins ratios for Rep-A30A′ could be expressed relative to those determined for Rep-WT. Results in Figure 5 showed that the NS2A-A30A′ mutation present in Rep-A30A′ indeed decreased both, β-gal/NS3 and β-gal/NS5 ratio by 20%∼40% relatively to Rep-WT and this trend was observed at all time-points measured. Interestingly, these results are in line with the frequency of PRF events reported for WT WNV_KUN_ virus [23].

We also determined the effect of PRF on viral proteins expression in WNV_KUN_-infected cells. However, during viral infection structural proteins assemble into viral particles that are secreted into media during viral secretion. This is likely to reduce the amount of intracellular structural proteins available in cell lysates and potentially skew intracellular structural/NS proteins ratio. To inhibit virion secretion, cells were treated with metabolic inhibitor brefeldin A (BFA) [36]. The addition of BFA late in the infection drastically reduces the amounts of secreted structural proteins (virions) without affecting viral protein synthesis [36] and hence it should reduce the effect of secretion on ratio of structural to non-structural proteins.

BHK-21 cells were infected with WNV_KUN_ WT and A30A′ viruses and at 48 and 72 hours post infection metabolic labelling with ^35^S methionine-cysteine in the presence or absence of BFA was performed. After 2 hours of labelling and 4 hours of chase the cell monolayers were harvested and lysed in RIPA buffer. Radiolabelled lysates were then immuno-precipitated with anti-E and anti-NS5 monoclonal antibodies and precipitated proteins were separated by SDS-PAGE and visualized after exposure to a phosphor-imaging screen. Again, quantification of the proteins of interest and calculation of E/NS5 ratios indicated that the A30A′ mutation was associated with a decrease in the E/NS5 ratio as compared to WT WNV_KUN_ infection (Figure 5C). This reduction was more evident in cells treated with BFA, indicating that virus secretion depleted viral structural proteins within the infected cell, as expected. These results indicate that the presence of PRF in the NS2A gene of WNV alters the equimolar ratio of viral structural and NS proteins, in the replicon or virus infection settings, demonstrating a role for PRF in translational regulation of viral protein synthesis.

#### PRF-deficient WNV_NY99_ virus is slightly attenuated in birds

**Figure 6:**
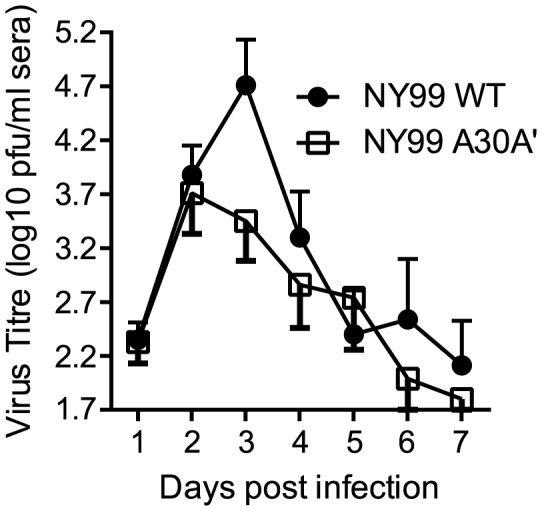
Viremia profiles of HOSPs inoculated with WNV_NY99_ WT (NY99 WT) or WNV_NY99_ A30A′ (NY99 A30A′) viruses. Groups of ten HOSPs were infected with 1500PFU of each virus and bled daily for 7 days after infection. Virus titres in serum were determined by plaque assay on BHK cells and plotted using GraphPad PRISM version 6.0a software. Error bars represent standard errors of the mean.

Birds serve as natural reservoir vertebrate hosts for WNV. Specifically, house sparrows (HOSPs) have been used previously as a natural avian host in order to assess genetic determinants of host competence as representative WNV avian model hosts [33], [37]–[39]. We inoculated groups of ten HOSPs with 1500 PFU of WT WNV_NY99_ and A30A′ mutant WNV_NY99_ viruses and collected serum daily for 7 days after infection to analyze virus titers by plaque assay on BHK cells. WT WNV_NY99_ virus reached peak average titers of 4.4 log_10_ PFU at day 3, while A30A′ mutant WNV_NY99_ virus reached an average titer of 3.7 log_10_ PFU at day 2 after infection (Figure 6). WT WNV_NY99_ titers remained higher than A30A′ WNV_NY99_ virus titers until day 4 after infection and both viruses were then essentially cleared by 7 dpi (Figure 6). It should be noted that significant variations in susceptibility to WNV infection are common between individual wild-caught HOSPs [40]. Although the differences in virus titres between WT and A30A′ WNV_NY99_ infections did not reach statistical significance by two way ANOVA comparison, the A30A′ WNV_NY99_ virus nevertheless showed noticeable attenuation at the peak time of infection (Figure 6), suggesting a role for PRF/NS1′ in WNV_NY99_ virulence in avian hosts.

### PRF-deficient WNV_KUN_ viruses show reduced replication and spread in *Culex annulirostris* mosquitoes

**Figure 7:**
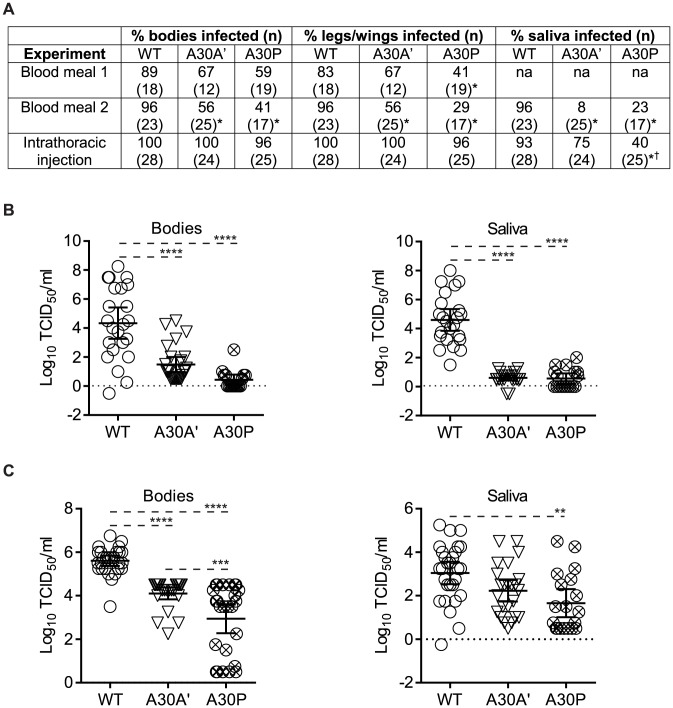
PRF/NS1′-deficient WNV_KUN_ mutants show reduced replication and dissemination in *Culex annulirostris* mosquitoes. (**A**) Infection (bodies), dissemination (legs and wings) and transmission (saliva) rates in mosquitoes exposed to either WT virus or mutant viruses by feeding on virus infected blood meals containing approximately 10^7^TCID_50_/mL of virus or intrathoracic inoculation with 220 nL of a 10^6^ TCID_50_/mL virus stock. A two-tailed Fisher's exact test was used to determine significant difference between infection, dissemination and transmission rates between each of the mutant viruses and the WT virus (*P<0.05) and between the mutant viruses (^†^P<0.05). (**B**) and (**C**) Mean ± 95% CI (confidence interval) virus titers in bodies and saliva expectorates from mosquitoes exposed to the mutant or WT-viruses by oral feeding (B) or intrathoracic inoculation (C). Twelve days post exposure, mosquitoes were processed by removing the legs and wings, and collecting the saliva using a forced salivation technique. Homogenized and filtered mosquito components (bodies, legs and wings, and saliva expectorates) were inoculated onto confluent monolayers of C6/36 cells in a 96 well microtiter plate, and infection detected by a cell culture enzyme immunoassay (CC-EIA). Viral titers of the bodies and saliva expectorates was determined by inoculation of ten-fold dilutions of the filtered homogenates in C6/36 cells with virus detected using the CC-EIA. A one-way ANOVA was employed to determine significant differences in mean viral titers within the mosquito bodies and saliva expectorates (****P<0.0001; ***P<0.001; **P<0.005; *P<0.05; only statistically significant differences are shown).

To examine the role of PRF/NS1′ on replication and transmission in mosquitoes, *Cx. annulirostris* were exposed *per os* to infectious blood meals containing approximately 10^7^ TCID_50_/mL of each of the WT, A30A′ and A30P WNV_KUN_ viruses in two separate experiments. The ability for the virus to infect and disseminate from the midgut was assessed in both experiments, while the ability for mosquitoes to transmit the viruses was also assessed in experiment 2. In both experiments, the body infection rates were higher in the mosquitoes exposed to WT WNV_KUN_ viruses than either of the mutant WNV_KUN_ viruses, with the difference being significant (*P*<0.001) in experiment 2 (Figure 7A). Similarly, the virus disseminated at lower rates in the mosquitoes exposed to the A30A′ and A30P viruses than the WT virus, with the difference not being significant (*P* = 0.3915) for A30A′ in experiment 1 only. Infection and dissemination rates were lower in mosquitoes exposed to A30P than those exposed to A30A′, although the difference was not significant. Finally, both the A30A′ and A30P viruses were transmitted at significantly lower rates (*P*<0.005) than the WT virus. The lack of PRF/NS1′ affected the ability for the virus to replicate in the mosquito body and the quantity of virus expectorated, with the titers in the bodies and saliva expectorates being significantly lower (*P*<0.0001) in mosquitoes infected with A30A′ and A30P, than those in the WT viruses (Figure 7B).

We also examined the ability for the virus to replicate in and be transmitted by *Cx. annulirostris* after IT inoculation, which is a mode of infection that bypasses the midgut of the mosquito. Bypassing the midgut produced higher rates of infection with the mutant viruses, with almost all mosquitoes becoming infected, irrespective of the virus inoculated (Figure 7A). Despite the high rates of infection for the 3 viruses, the titers of virus within the bodies were significantly lower in the mutant viruses than the WT virus (Figure 7C). This potentially led to the lower transmission rate observed in the mutant viruses when compared to the WT virus, with the rate being significantly lower in mosquitoes inoculated with A30P (Figure 7A).

## Discussion

A novel translational mechanism for the synthesis of the NS1′ protein by encephalitic flaviviruses from the Japanese encephalitis serogroup was recently predicted by computer modelling [22] and then demonstrated experimentally for WNV_KUN_ [23]. The uniqueness of NS1′ expression by encephalitic flaviviruses had sparked significant interest in NS1′ functions related to viral virulence. Indeed, recent reports had focused on the potential functions of the NS1′ protein in mammalian cell systems and mouse models of disease and identified several potential functions for NS1′ protein including RNA replication, virion formation, and neurovirulence [23], [24], [26], [28], [30].

Data presented in this manuscript extended our previous knowledge of PRF/NS1′ and its role(s) during infection of mammalian, avian, and mosquito cells. WT and A30A′ replicons and viruses accumulated at similar levels in different cells suggesting that the presence or absence of PRF/NS1′ does not affect the rates of viral RNA synthesis or virion accumulation and/or spread from cell to cell. This is in contradiction to the findings of Winkelmann et al. who found that abolishment of PRF and NS1′ production impacted the ability of the mutated replicon to be encapsidated into virus-like particles [26]. However, it is possible that the effect observed in those experiments was due to the use of a heterologous trans-encapsidation model where WNV replicon RNAs were packaged by dengue virus structural proteins since when WNV structural proteins were used for packaging WNV replicons, the difference in packaging efficiency between PRF-competent and PRF-deficient replicon RNAs was greatly reduced [26].

Our transcriptome analysis of WT and IRF3^−/−^/7^−/−^ MEFs upon infection with WT and PRF-deficient viruses (A30A′ and A30P) provided a detailed overview of host gene expression changes after infection and a guide for future investigations. Changes in the global gene expression induced by infections with WT or A30A′ viruses were similar with only a small number of genes differentially expressed and also with relatively small differences in their levels of expression. These differentially expressed genes included transcription factors, protease inhibitors, and ER stress response elements. Importantly however, all of these differences despite being relatively small were highly significant indicating that even relatively small changes in gene expression may have biological significance. Further studies should focus on determining the role for each of these differentially expressed host genes in WNV-host interactions in vivo.

In contrast to small differences observed between WT and A30A′ viruses, significantly higher number of differentially expressed transcripts and higher fold changes for them were found between WT and A30P or A30A′ and A30P infections, suggesting that the Ala to Pro substitution in the NS2A protein has a more dramatic effect than PRF alone on the host response to infection. These differentially expressed genes in response to A30P infection include transcription factors, anti-apoptotic signals, and thermo-tolerance factors. Further studies are required to determine the role for the host genes shown the most differences in induction/repression between WT and A30P or A30A′ and A30P viruses in WNV-host interactions both in vitro and in vivo.

Variant analysis of virus populations by Ion Torrent sequencing detected a significant population of genomes in the WT swarm that contained PRF-abolishing mutation at position 3615 not present in the WT genomic sequence (proportion column in Table 5, 0.889 = ∼11%). On the other hand, the A30A′ swarm did not contain mutations to revert to WT sequence at position 3615 (proportion column, 0.999 = 0.1%) indicating that A30A′ had lost the need and/or ability to revert to WT through “error-prone” replication events. The significance of the 3615 position, and the PRF events, was underscored by our observations in position 3618. In our initial study [23], we introduced the extra G to A change at position 3618 of the A30A′ mutant with the purpose of minimizing a chance of reverse mutation(s) back to the WT sequences; this position *per se* does not play a role in pseudoknot formation and PRF. In contrast to the ∼11% of WT population reads containing a mutation at position 3615 less than 1% of reads showed mutations at position 3618. This suggest the intriguing possibility that during WT infection a significant proportion of the genomes produced (∼11%) are PRF-deficient, a de-facto compensation for at least a proportion of the −1 programmed ribosomal frameshifting events occurring during WT infection [23]. Further studies will be required to elucidate the true meaning of the existence of this virus sub-population, however it is likely that regulation of PRF frequency is employed by the virus to maintain the right balance between higher and lower replication efficiencies as the result of more or less efficient PRF event.

We demonstrated that PRF altered the levels of structural and non-structural proteins synthesis creating a *de-facto* over-expression of structural proteins over non-structural proteins; to our knowledge, this is the first time that the effect of the PRF on the expression of flavivirus proteins has been demonstrated. We hypothesize that such “excess” of structural proteins may lead to an increase in prM-E particles and/or act as an elegant modulator of viral protein expression, which may indirectly regulate host responses to viral infections. Pseudoknot formation and −1 ribosomal frameshifting in coding regions of viral genomes have been described previously [41], [42]. In retroviruses, precise ratios of frameshifting allows expression of the viral Gag-Pol polyprotein and sets an optimal Gag∶Gag-Pol ratio for virion assembly and packaging of reverse transcriptase [43]. Similarly, in SARS-CoV pp1a and pp1ab, which are expressed by frameshifting, are predicted to form a heterodimer with a stoichiometry of 8/1 as part of the replication machinery [44], [45]. In both examples maintaining precise ratios of frameshifting is crucial for virus accumulation and we would argue that similar mechanisms might be required during infection of mosquitoes and birds with WT and PRF-deficient WNVs. Indeed, we demonstrated that PRF affects virus infectivity and/or spread in mosquito vectors and in the generation of elevated viremias in an avian host species, HOSPs. PRF-deficient mutant (A30A′) showed lower titers in birds and mosquitoes and also reduced ability to be transmitted by mosquitoes as shown by lower percentage of infected saliva for these mutants. A role in replication of PRF/NS1′ in birds and mosquitoes would explain the phenotypes observed despite this and other reports where differences in replication in several cells lines infected with PRF-deficient mutants [23], [30] were not observed. The difference between *in vitro* and *in vivo* results is often seen for the bird phenotype [39]. Interestingly, in contrast to our results in avian DF-1 cells, JEV PRF mutant virus replicated significantly less efficiently than wild type JEV virus in DF-1 cells [25], suggesting that difference exists also between different albeit closely related flaviviruses in their ability to replicate in the absence of PRF *in vitro*.

It is important to note that upon viral infection, vertebrates establish an antiviral state mostly through type I interferon responses [46], [47] while insects respond primarily through RNA interference (RNAi) [48], [49] or the activation of immune pathways, including Toll, Imd and JAK/STAT pathways [50]. Our data suggest the existence of interactions between NS1′ protein (or an effect of PRF) and host response pathway(s)/factors that are efficient (present) in whole mosquitoes but not in cellular models of infection, including mosquito cell lines, some of which are known to be deficient in a host factor, e.g. C6/36 cells are deficient in dicer 2 [51]. In this context, the role of insect gut barriers and its interaction with the flavivirus genome is critical. Increased viral accumulation in the gut has been reported to correlate with the ability of the arboviral infection to spread throughout the mosquito and determine its capacity to transmit infection by bite [52]. Efficient infection of the midgut is likely to trigger a robust innate response by the host including, but not limited to, stimulation of enzymes involved in the oxidative stress response and re-epithelialization of damaged cells of the midgut wall [53], [54]. It is intriguing that the PRF motif is highly conserved among the JEV serocomplex viruses that utilize both *Culex* mosquito vector and avians as their primary vertebrate replication hosts while other flaviviruses that utilize primate and *Aedes* vectors such as Dengue virus 1–4 and Yellow fever viruses or rodents and ticks have not demonstrated to encode PRF. The results presented herein show the role of PRF as mechanism for modulation of the stoichiometric ratio of structural to non-structural genetic determinants that could be involved in modulating mosquito RNAi responses or potentially avian innate immune responses.

## Materials and Methods

### Cells and viruses

BHK, Vero76, LLC-MK2 and DF-1 and MEF cells were propagated and maintained in Dulbecco's Modified Eagle Medium (DMEM, Gibco) containing 5% fetal calf serum (FCS), 50 U/ml penicillin, 50 µg/ml streptomycin, 2 mM glutamax and 10 mM HEPES at 37°C and 5% CO_2_.

KUN WT, A30A′, and A30P viruses were obtained by in vitro transcription of corresponding DNA plasmids [23] and electroporation of viral RNA transcripts into BHK cells. NY99 WT and A30A′ viruses were generated from in vitro ligated two plasmid system [55]. Passage 0 viruses were harvested 1–3 days post electroporation, titrated by plaque assays, and used at noted multiplicity of infection (MOI). Mutations introduced into mutant viruses and replicons were confirmed by RT-PCR and sequencing from viral and replicon RNAs.

### In vitro transcription and electroporation

Linearized DNA template from KUNV replicon plasmids was transcribed using SP6 RNA polymerase (Roche) as per manufacturer instructions and the resulting KUN replicon RNA transcripts were electroporated into BHK cells (Bio-Rad GenePulser II apparatus; 25 µF capacitance, 1.5 kV voltage, infinite resistance, two pulses with 10 second interval, with the optimal time constant between 0.7 and 0.8 ms). Electroporated cells were then resuspended in 10 ml of DMEM containing 5% FCS, seeded into culture plates, and incubated at 37°C under 5% CO_2_ and humid conditions. 72 h post electroporation (hpe) total cellular RNA was extracted using Trizol reagent (Invitrogen) following manufacturer instructions and separated in a denaturing 1% agarose gel. After transfer of total RNA onto nitrocellulose membrane viral genome-specific RNAs were detected by hybridization with P^32^-labelled KUNV 3′-UTR-specific probe.

For in-situ X-gal staining replicon-transfected cells in a 24-well plate were washed with PBS, fixed with 500 µl 4% paraformaldehyde for 20 min at room temperature and washed with 500 µl PBS 3 times. B-gal activity was detected by adding 200 µl of X-gal stain solution (50 µl K_4_Fe(CN)_6_-3H_2_O, 50 µl K_3_Fe(CN)_6_, 2 µl 1 M MgCl_2_, 50 µl X-gal stock(20 mg/ml) and 850 µl PBS) to each well, and incubating at 37°C for 1 h.

### Immunofluorescence (IFA) analysis

Vero 76 and BHK cells were seeded into 24-well plates and infected at MOI 0.01. Infected cells were fixed and permeabilised (4% paraformaldehyde 20 min, 0.1% Triton X-100 for 10 min) at 24, 36, 48, 60 hours post infection and stained for 1 h at room temperature with mouse anti-E monoclonal antibodies 3.91D [56], followed by 30 min staining with the secondary Alexa Fluor 488 goat anti-mouse antibodies and DAPI (nuclear stain, Sigma-Aldrich), with intercalating 3×5 min washes with PBS between each step. After a final wash with PBS, cells were imaged using IN-cell 1000 analyser (GE Healthcare) at the required excitation and emission wavelengths and the percentage of E-positive cells (% infected) was determined using IN-Cell 1000 Workstation software where: % infected cells = [(number of cells at 480 nm)/(number of cells at 360 nm)]_KUNV_×100−[(number of cells at 480 nm)/(number of cells at 360 nm)]_mock_×100.

### Growth curves and cytopathicity (CPE) assay

DF-1 and LLC-MK2 cells were seeded into 6-well plates and infected with WT, A30A, and A30P viruses at MOI of 1. Culture supernatant was harvested and viral tiers were determined by plaque assay [55] at 0, 42, 66, and 90 hpi. CPE was analysed by seeding DF-1 or LLC-MK2 cells into a 24-well plate and infecting at MOI 1. At 5 dpi cells were fixed with 4% formaldehyde for 1 h and stained with 0.2% Crystal Violet for 20 min. Images of CPE staining were obtained using Epson perfection V700 photo scanner.

### Microarray analysis

Low passage primary WT and IRF3^−/−^/7^−/−^ MEFs were infected with WT, A30A′, or A30P viruses at MOI of 1. At 48 hpi total RNA was extracted with Trizol and an Illumina DNA microarray was run on 16 samples (8 experimental samples with duplicates). The raw signal intensity was extracted from the encrypted binary.idat files using the IDATreader package (version 0.2.0). The raw data were processed to stabilise the variance [57] and the Quantile normalization methods within the Limma package of Bioconductor were implemented. In addition to the normalization of the experimental data, an averaging of the duplicated array probes was performed so the number of analytical features decreased to the number of uniquely identified probes. The statistical analysis of the differences in gene expression pattern were again performed by methods implemented within the Limma Bioconductor package and differential expression was defined when a gene had a log-fold-change of <−1 or >1. The expectation value for maximum accepted error corrected probability was defined as 0.05 and error correction for the p-values was performed according to methods described by Benjamini and Hochberg [58].

### Variant analysis by Ion Torrent sequencing

The quality and characteristics of the Ion torrent sequences from WT and A30A viruses, in FASTQ format, was investigated using FASTQC. The distribution of the base quality scores across the reads were typical for Ion Torrent data and in both samples the median sequence length was approximately 220 nucleotides in length. The Ion Torrent FASTQ format DNA sequence reads were mapped onto the reference genomes (WT and A30A′) using the bwa software and an indexed BAM file was prepared using the Picard software. Review of the BAM alignment using Tablet revealed that the bulk of the reference sequences were covered by between 3000× to 6000× genome coverage and only a very small fraction of the reference sequence was not sampled to at least 50× coverage. A manual review of the sequence alignment was performed and identified single nucleotide deletion events occurring in both genomes but only present in the forward strand were excluded from further analysis; these deletion variants were deemed a consequence of the Ion Torrent sequencing technology.

### Protein analysis

Cells were lysed with RIPA buffer and with NuPAGE LDS sample loading buffer (Invitrogen) for 20 min and heated at 95% for 3 min before loading total protein samples (∼10 µl) onto a 15-well precast Express PAGE gel (Genescript). The proteins were separated by electrophoresis at 120 V for 1 h and transferred to PDVF membrane (GE Healthcare) for 1.5 h at 45 V using a wet transfer apparatus (BioRad). Membranes were blocked for 1 h in 5% non-fat milk (BioRad) and then probed for 1 h at room temperature with gentle rocking with primary antibodies diluted in 5% non-fat milk. The membranes were washed 3 times with TBST (at least 5 min each time) and incubated for 1 h with secondary antibodies diluted in PBS. After a final wash with TBST, the blots were developed by LI-COR Odyssey Infrared Imaging System (LI-COR Biosciences) and integrated intensity of each band was used for calculating the ratios. Ratios of structural (or reporter β-gal activity) vs non-structural proteins observed during WT virus infections or WT replicon RNA replication were set to 1 and this was used as the reference for calculating corresponding ratios in PRF-deficient virus or PRF-deficient replicon replication.

### Characterization of NY99 WT and A30A′ viruses in house sparrows

House sparrows (HOSPs) were collected under approved animal care and use protocols and field studies did not involve endangered or protected species. All animal studies presented herein were approved by Institutional Animal Care and Use Committees at the Division of Vector-Borne Diseases, Centers for Disease Control and Prevention (approval number 13-009). All protocols and practices for the handling and manipulation of sparrows were in accordance with the guidelines of the American Veterinary Medical Association (AVMA) for humane treatment of laboratory animals as well as the “Guidelines to the Use of Wild Birds in Research” published by the ornithological council 3^rd^ edition (2010).

HOSPs were trapped by seed-baited ground traps in Larimer County, Colorado. Birds were bled and sera assayed for neutralizing antibodies against WNV and SLEVs as previously described [32]. Ten WNV/SLEV seronegative HOSPs were inoculated subcutaneously in the breast region by 28-gauge needle with 1500 PFU of the WT and A30A′ viruses. Viral inocula were diluted in PBS (0.1 mL final volume). Inoculated HOSPs were bled once daily by jugular venipuncture through 7 dpi. 0.1 mL of whole blood was drawn and expelled into a cryovial containing 0.45 mL of BA-1 media. Samples were allowed to clot at room temperature for >20 min and spun at 3500-g for 10 min. The 1∶10 sera sample dilutions were frozen at −80°C until titrated by plaque assay on BHK clone 15 cells for infectious titer determination as previously described [32]. The virus titres were plotted using GraphPad PRISM version 6.0a software and statistical analyses was performed using two-way ANOVA comparison using the same software package.

### Characterization of wild-type and mutant KUN viruses in mosquitoes

We exposed *Culex annulirostris* mosquitoes to WT, A30A, and A30P viruses via infectious blood meal or intrathoracic (IT) inoculation. These mosquitoes were colonized from material collected from the Boondall Wetlands, Brisbane, Australia in 1998 and have been maintained in a colony by the Australian Army Malaria Institute for >100 generations. Previous experiments had demonstrated high infection and transmission rates with a wild type WNV_KUN_ strain in this colony of *Cx. annulirostris* [59].

For virus exposure by blood feeding, 5–10 days old mosquitoes were allowed to feed on a blood meal consisting of WT or mutant viruses diluted in commercially available defibrinated sheep blood using the pledget method of Wells [60]. We also explored accumulation and spread of the WT and mutant viruses following IT inoculation. IT inoculation bypasses the midgut and associated barriers while also allowing for standard amounts of virus to be delivered directly producing a more accurate determination of the replication abilities of the virus within the mosquito [61]. For IT inoculation, 5–10 day old mosquitoes were inoculated with approximately 220 nl of virus suspension.

Following feeding or inoculation, mosquitoes were maintained on 10% sucrose at 28°C, high relative humidity and a 12L∶12D lighting regime in an environmental growth chamber. After 12 days incubation, mosquitoes were processed to determine infection, dissemination and transmission rates. Mosquitoes were anaesthetized with CO_2_ gas and the legs and wings removed. Detection of the virus in the legs and wings indicates that the virus has escaped from the midgut and has disseminated through the hemocoel [52]. To assess transmission potential, saliva was collected by inserting the proboscis of the mosquito into a capillary tube containing 25 µL of growth media, supplemented with 20% FCS. Following transmission attempts, the saliva expectorate were placed in 2 ml vials containing 0.6 ml of growth media with 3% FCS, and the bodies, and legs and wings were placed in separate 2 ml vials containing 1 ml of growth media with 3% FCS and three sterile 5 mm glass beads. All samples were stored at −80°C prior to analysis.

The bodies, and legs and wings were homogenized using a SPEX 8000 mixer/mill (Spex Industries, Edison, NJ). All samples were filtered through a 0.2 µm Supor membrane filter (Pall Corporation, Ann Arbor, MI) to reduce bacterial and fungal contamination. The filtrates were inoculated into the wells of a 96-well microtiter plate seeded with confluent C6/36 (*Aedes albopictus*) cell monolayers. Plates were incubated at 28°C in the absence of CO_2_. To determine the virus titers, the bodies and saliva expectorates were titrated as ten-fold dilutions, whilst the legs and wings were assessed for infection only. After 7 days, plates were fixed with PBS/acetone and virus infection detected by a cell culture enzyme immunoassay [62] with the monoclonal anti-E antibody 3.91D [56].

### Statistical analysis of mosquito data

For the characterization of the wild type and mutant viruses in *Cx. annulirostris*, body infection rate was defined as the number percentage of mosquitoes containing virus in their bodies (number positive/number tested). The disseminated infection rate was defined as the percentage of mosquitoes containing virus in their legs and wings (number positive/number tested). The transmission rate was defined as the percentage of mosquito expectorates in which virus was detected (number of positive expectorates/number tested). Infection, dissemination and transmission rates were analysed using Chi-square and Fisher's exact test. Differences in viral titers within mosquito bodies and saliva expectorates were analysed using multiple factor ANOVA test and homogeneity of variance was performed using Bartlett's test. P values<0.05 were considered statistically significant. All analyses were performed using R and/or GraphPad statistical packages.
